# pH sensing and regulation in cancer

**DOI:** 10.3389/fphys.2013.00370

**Published:** 2013-12-17

**Authors:** Mehdi Damaghi, Jonathan W. Wojtkowiak, Robert J. Gillies

**Affiliations:** Department of Cancer Imaging and Metabolism, Moffitt Cancer Center and Research InstituteTampa, FL, USA

**Keywords:** proton sensors, pH regulators, cancer microenvironment, buffer therapy, extracellular acidification, intracellular pH

## Abstract

Cells maintain intracellular pH (pH_i_) within a narrow range (7.1–7.2) by controlling membrane proton pumps and transporters whose activity is set by intra-cytoplasmic pH sensors. These sensors have the ability to recognize and induce cellular responses to maintain the pH_i_, often at the expense of acidifying the extracellular pH. In turn, extracellular acidification impacts cells via specific acid-sensing ion channels (ASICs) and proton-sensing G-protein coupled receptors (GPCRs). In this review, we will discuss some of the major players in proton sensing at the plasma membrane and their downstream consequences in cancer cells and how these pH-mediated changes affect processes such as migration and metastasis. The complex mechanisms by which they transduce acid pH signals to the cytoplasm and nucleus are not well understood. However, there is evidence that expression of proton-sensing GPCRs such as GPR4, TDAG8, and OGR1 can regulate aspects of tumorigenesis and invasion, including cofilin and talin regulated actin (de-)polymerization. Major mechanisms for maintenance of pH_i_ homeostasis include monocarboxylate, bicarbonate, and proton transporters. Notably, there is little evidence suggesting a link between their activities and those of the extracellular H^+^-sensors, suggesting a mechanistic disconnect between intra- and extracellular pH. Understanding the mechanisms of pH sensing and regulation may lead to novel and informed therapeutic strategies that can target acidosis, a common physical hallmark of solid tumors.

## Acidosis and pH regulation in cancer cells

**Figure 1:**
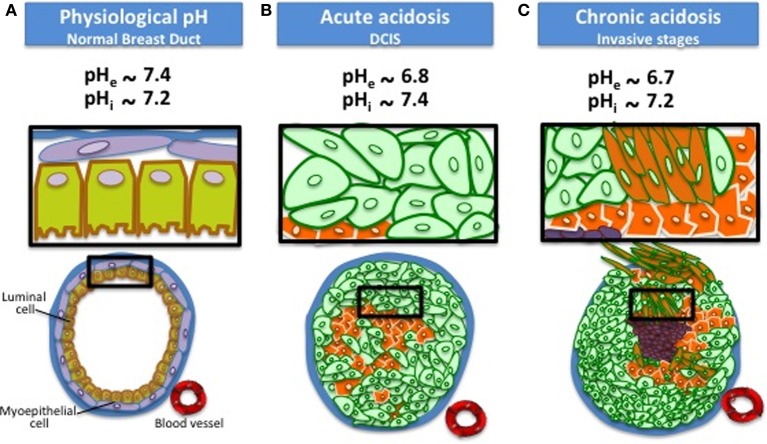
**Reversed extracellular and intracellular pH in cancer cells compared to normal cells**. Cancer cells have a reversed pH gradient compared with normal differentiated cells that is cancer cells have a higher pH_i_ and a lower pH_e_ than normal cells in acute acidosis conditions. The pH_e_ becomes even lower (~6.7) in chronic acidosis. This disruption facilitates the adaptive behaviors of cancer cells such as cytoskeleton remodeling and directed migration, apoptosis evasion, extracellular matrix (ECM) remodeling, invasion, and metastasis.

The physical microenvironment of solid tumors is heterogeneous. A combination of poor vascular perfusion, regional hypoxia, and increased flux of carbons through fermentative glycolysis leads to extracellular acidosis in solid tumors; with extracellular pH (pH_e_) values as low as 6.5 (Hashim et al., 2011). Notably, environmental acidification initially occurs early in cancers, during the avascular phase of carcinoma *in situ*, CIS (Barathova et al., 2008). There is reason to suspect that the most acidic CIS are more prone to progress to local invasion. With intraductal hyperplasia, IDH, cancer cells become increasingly distant from the basement membrane and underlying vasculature. These cells encounter an oxygen poor environment and, through a Pasteur effect, undergo a metabolic switch toward a more glycolytic phenotype (Gatenby and Gillies, 2004). Consequently, the proton (H^+^) concentration increases within the lumen due to diffusion limitations and increased production of acid from hypoxic-glycolytic cells, causing the interior of the lumen to become highly acidic. Later on in carcinogenesis, the glycolytic phenotype can become “hardwired” (i.e., the Warburg effect) and this leads to the continued generation of metabolic acids, even in well-oxygenated conditions (Figure 1). Adaptations to this highly acidic microenvironment are critical steps in transition from an avascular pre-invasive tumor to a malignant invasive carcinoma that has access to patent vasculature (Lee et al., 2008). If acidosis contributes to the development of invasive carcinoma, then an intervention that neutralizes pH_e_ will either delay or inhibit this last step in carcinogenesis. We have shown, using 31P-MRS of 3-aminopropylphosphanate, that administration of oral buffers (200 mM sodium bicarbonate) significantly raises the pH_e_ of xenograft tumors without altering pH_i_ (Raghunand et al., 1999). More importantly, buffer therapy has the ability to reduce the formation of spontaneous and experimental MDA-MB-231 lung metastases (Robey et al., 2009) and suppress the formation of spontaneous prostate tumors in the TRAMP prostate GEMM (Ibrahim-Hashim et al., 2012).

The glycolytic byproducts, lactate and H^+^, challenge the mechanisms to maintain intracellular pH (pH_i_) homeostasis. Intracellular acidification has been shown to be cytotoxic through induction of apoptosis (Gottlieb et al., 1996; Lagadic-Gossmann et al., 2004). To overcome low pH_i_ due to elevated rates of glycolysis, cancer cells employ a large redundancy of mechanisms to remove acids in order to maintain physiological pH_i_. In response to glycolytic acidosis, pH_i_ can be be maintained via lactate and H^+^ efflux by monocarboxylate transporters and Na-driven proton extrusion, respectively (Gillies, 2002; Gallagher et al., 2008). As a consequence, the pH of the extracellular space of tumors becomes acidic; forming a reversed pH gradient (pH_e_ < pH_i_) in comparison to normal physiological conditions (pH_e_ > pH_i_) (Figure 1) (Stubbs et al., 1994; Webb et al., 2011).

A reversed, acid-outside, pH gradient promotes cancer progression, via the relatively acidic pH_e_ that induces migration and invasion (Bradley et al., 2011; Hanahan and Weinberg, 2011). Increased pH_i_ by itself also has effects on cancer cell function such as increased proliferation (Moolenaar et al., 1986), promoting cell survival by limiting apoptosis, which is associated with intracellular acidification (Matsuyama and Reed, 2000), and selective advantages of growth-factor independent proliferation.

In order to survive the dynamic nature of pH in tumors, both cancerous and normal stromal cells require the ability to sense minute pH changes and respond appropriately to maintain pH_i_ homeostasis. One mechanism by which pH sensing occurs is through post-translational protonation of amino acid side chains, in particular the imidazole side chain of histidine residues. Although under-represented in the human proteome with ~2% frequency (Webb et al., 2011), histidines are found in critical regions of G-protein coupled receptors, GPCRs (OGR1 and GPR4), intracellular molecules involved in actin assembly (Talin and Cofilin), membrane proton pumps and acid-sensing ion channels, ASICs (Schonichen et al., 2013b). Protonation and de-protonation has been experimentally shown to change protein structure and thus, alter protein-protein binding affinity, change protein stability, modify protein function, and alter subcellular localization (Schonichen et al., 2013b). Evolutionarily, histidines must confer some selective advantage for cancers, as 15% of the 2000 identified somatic mutations in cancer involve histidine subsitutions, with Arg-to-His being the most frequent (Kan et al., 2010). The pH sensing role of histidines in cancer will be discussed further in this review.

## Section 1: the intracellular pH

**Figure 2:**
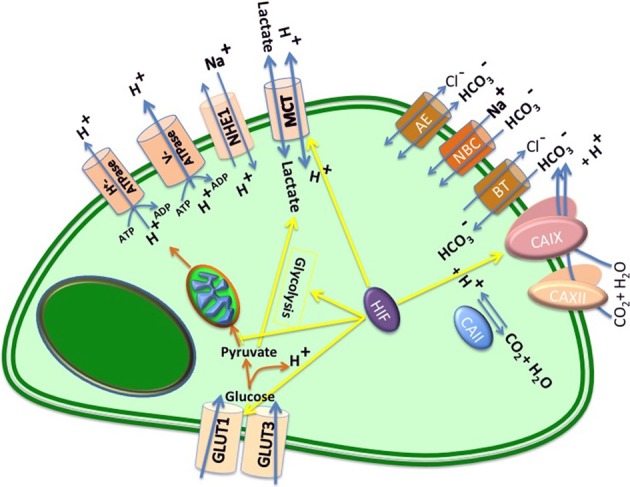
**Major pH regulators in a cancer cell**. After glucose uptake by specific transporters (GLUT1 and GLUT3), glucose is converted to pyruvate, generating 2 ATP per glucose and proton. Based on Pasteur effect, in the presence of oxygen, pyruvate is oxidized to HCO^−^_3_, generating 36 additional ATP per glucose; in the absence of oxygen pyruvate is reduced to lactate, which is exported to extracellular space. However, as Warburg proposed glycolysis is potent in cancer cells. Notably both processes produce protons (H^+^), which cause acidification of the extracellular space. This figure represents main proteins that regulate intracellular and extracellular pH in tumors, including: monocarboxylate transporters (MCTs), which transport lactic acid and other monocarboxylates formed by the glycolytic degradation of glucose; the plasma membrane proton pump vacuolar ATPase (V-ATPase); Na^+^/H^+^ exchangers (NHEs); anion exchangers (AEs); carbonic anhydrases (CAII, CAIX, and CA XII); Na^+^/HCO^−^_3_ co-transporters (NBCs), and HCO^−^_3_-transporters (BTs).

Regulation of pH starts with changes in the expression or activity of several plasma membrane molecules such as pumps and transporters that facilitate H^+^ efflux to maintain the alkaline pH_i_ and the acidic pH_e_ in tumor cells (Huber et al., 2010). These redundant mechanisms of tumor pH regulation include: Carbonic Anhydrases such as CA2, CA9, and CA12 (Barathova et al., 2008; Supuran et al., 2010); V-ATPase (Hinton et al., 2009); Na^+^/HCO^−^_3_ co-transporters (Pouyssegur and Mechta-Grigoriou, 2006); the Na^+^-driven Cl^−^/HCO^−^_3_ exchanger, SLC4A8 (Pouyssegur et al., 2006); the monocarboxylate transporters: MCT1, MCT2, MCT3, and MCT4 (Enerson and Drewes, 2003); Na^+^/H^+^ exchanger 1, NHE1, which is also known as SLC9A1 (Amith and Fliegel, 2013); and the anion exchangers AE1 and AE2, also known as SLC4A1 and SLC4A2 (Barneaud-Rocca et al., 2011) (Figure 2).

### Carbonic anhydrases (CAs)

The CA family of proteins are metalloenzymes that catalyze the conversion of carbon dioxide generated in high amounts as the final product of oxidative phosphorylation and water to bicarbonate and protons. A primary function of CAs is to maintain the intra- and extracellular acid–base balance. CAIX and CAXII are transmembrane CAs that have been identified to play roles in tumor progression and metastasis (Tafreshi et al., 2012; Ilie et al., 2013). It has recently been shown that CAIX in colon cancer and glioblastoma promotes tumor growth and necrosis (McIntyre et al., 2012). As a transcriptional target of HIF1α, CAIX expression is upregulated in hypoxic regions of tumors including breast cancer (Tafreshi et al., 2012). CAXII is also overexpressed in tumors and is associated with cancer progression. Although CAXII and CAIX can be expressed in the same cells, their intratumoral distributions are often different with more global and more regional distributions, respectively (Tafreshi et al., 2012). Intracellular CAs (e.g., CAII) will dehydrate metabolically produced bicarbonate into aqueous CO_2_ in a reaction consuming a proton. By mass action, the CO_2_ is exported across the bilayer or through aquaporins, where it is re-hydrated by CAIX or CAXII to re-produce HCO^−^_3_ in a reaction producing an extracellular proton. The HCO^−^_3_ can then diffuse out of the tumor, or re-enter the cell via bicarbonate transporters or anion exchangers to maintain a more alkaline pH_i_ (Xie et al., 2009; Swietach et al., 2010). CAIX is an important facilitator of acid diffusion that its importance in regulating tumor pH has made it a potential target for designing new therapeutics against cancer (Swietach et al., 2010).

### HCO^−^_3_ transporters

These proteins are mainly clustered into three classes: electroneutral Cl^−^/HCO^−^_3_ exchangers of the SLC4 family; the NBC family of Na^+^/HCO^−^_3_ co-transporters; and anion transporters of the SLC26 family. Additionally, HCO^−^_3_ transport via aquaporins has been reported (Cooper et al., 2002). These transporters facilitate the movement of HCO^−^_3_ ions across plasma membranes to either acidify or alkalinize the pH_i_ (Vilas et al., 2009). It has been shown via functional assays that the Na^+^/HCO^−^_3_ co-transporter (NBCn1) is the predominant mechanism for acid extrusion in breast carcinoma tissue when pH_i_ levels are greater than 6.6 (Boedtkjer et al., 2013). In fact, inhibition of NBCn1, in NHE1 expressing cells, resulted in pH_i_ acidification suggesting that NBCn1 should garner further attention as a therapeutic target (Boedtkjer and Aalkjaer, 2012; Boedtkjer et al., 2012).

### NHE1

NHE1 is the most common isoform of the Na^+^/H^+^ exchanger (NHE) family that is present in all mammalian cells. The NHEs (NHE1-NHE7) are amongst the most active transporters in pH_i_ homeostasis and use the powerful sodium electrochemical gradient to extrude H^+^ if the cytosolic pH becomes too acidic. Notably, there is a pH sensing site on the internal surface of the NHE that, if protonated, activates transport. This “set-point” can be altered by growth factor (e.g., EGF) activation. In cancer cells with their high metabolic acid load, NHE1 hyperactively extrudes protons into the extracellular space making it acidic. Particularly in breast cancer, it has been suggested that upregulation of NHE1 activity is a main factor for increased invasion and metastasis (Gatenby and Gillies, 2008; Busco et al., 2010; Amith and Fliegel, 2013). For example, it has been shown that CD44, which is a cell surface glycoprotein, is localized at the invadopodia of an invasive breast cancer cell line, MDA-MB-231 cells. CD44 stimulates NHE1 activity leading to invasion through the RhoA effector ROCK1 (Bourguignon et al., 2004). It has also been shown that RAC1 and the RhoA–ROCK–PI3K pathway are involved in CD44-induced cell invasion (Bourguignon et al., 2001). In cervical cancer, it has been shown that NHE1 up-regulation by EGF is important for cervical cancer cell invasiveness (Chiang et al., 2008). Hence, it appears that signaling molecules can activate NHE1 leading to a reversed pH gradient. Treating MDA-MB-231 cells with an NHE inhibitor (cariporide) suppressed the invasive capability of this highly metastatic breast cancer cell line (Lin et al., 2011a,b).

### V-ATPase

Vacuolar ATPases pump protons out of the cytoplasm and into intracellular vesicles, such as lysosomes in an ATP dependent process (Horova et al., 2013) and these events are also altered in cancer (Perez-Sayans et al., 2009a,b). Importantly, the activity of these pumps is tightly coupled to endosome trafficking, suggesting that they need not be resident at the plasma membrane to exert their pH regulatory effects (Martinez-Zaguilan et al., 1999, 2013). Indeed, Glunde et al. (2003, 2005) and Cardelli (Steffan et al., 2009) have both shown that low pH induces the turnover of endosomal-lysosomal compartments with the extracellular space, which could be an indirect mechanism for V-ATPase mediated extrusion of H^+^ from the cytosol. Saw et al. recently showed that high-metastatic B16-F10 melanoma cells overexpress the *a*3 isoform V-ATPase compared to the low-metastatic B16 parental cells and V-ATPase inhibitors reduced bone metastasis (Saw et al., 2011).

### Monocarboxylate transporter (MCTs)

MCTs transport mono-carboxylic acids (such as lactate, pyruvate, and ketone bodies) into and out of cells across plasma and mitochondrial membranes. The MCT family is composed of 14 members with similar topology and can be found expressed in a wide range of tissues. Only 4 isoforms (MCT1–MCT4) have been functionally characterized as proton-linked monocarboxylate transporters (Morris and Felmlee, 2008; Halestrap, 2012; Halestrap and Wilson, 2012). This is critical as MCTs (MCT1 and MCT4) are routinely overexpressed in tumors primarily regulating the efflux of lactate and protons as byproducts of glycolysis from intracellular to extracellular space in order to maintain physiological pH_i_ thus, contributing to extracellular acidosis. High expression levels of these transporters have been linked to poor prognosis and disease progression in colorectal, ovarian, gastric, and breast carcinomas (Pinheiro et al., 2009a,b, 2010; Chen et al., 2010).

So far, we have focused on tumor hypoxia and efflux mechanisms by which cancer cells regulate pH_i_ on account of increased glycolysis. One mechanism discussed was the outward flux of lactic acid by MCT1/4. We must appreciate, however, that it is not uncommon to observe well oxygenated and nutrient rich tissue coexisting with hypoxic and carbon poor tissue (Gatenby et al., 2013). Although these regions are metabolically distinct, it is hypothesized that carbon symbiosis occurs between glycolytic and oxidative compartments to promote tumor growth wherein hypoxic tissue mostly consumes glucose and oxygen rich tissue consumes the glycolytic byproduct, lactate (Nakajima and Van Houten, 2013). Sonveaux et al. have proposed that this transpires through specific intratumoral MCT1 expression patterns that are environmentally regulated (Sonveaux et al., 2008). Using human cervical and colon xenograft tumor models, MCT1 expression was highest in well-oxygenated tissue (CD31 positive) with little to no detectable expression in hypoxic (Pimonidazole positive) regions. The authors show that oxygenated tissue promotes the inward flux of lactate via MCT1 to fuel the TCA cycle and that *in vivo* inhibition of MCT1 with α-cyano-4-hydroxycinnamate limited tumor growth. Sonveaux hypothesized that the anti-tumor effect was a result of a metabolic switch from lactate-fueled respiration to glycolysis in oxygenated tissue further reducing the carbon source for hypoxic tissue (Sonveaux et al., 2008).

Similar observations were made by Koukourakis et al. in colorectal adenocarcinoma (Koukourakis et al., 2006). Using patient tissue, tumor, and stromal compartments were histologically and thus, metabolically distinguishable based on MCT and glucose transporter expression patterns. Colorectal adenocarcinoma was shown to express high glucose uptake (GLUT1) and lactate efflux (MCT1) proteins in respect to tumor associated stromal cells that expressed low glucose uptake and high lactate influx (MCT1 and MCT2) proteins driving lactate oxidation (Koukourakis et al., 2006). Similar cancer-stromal cell metabolic collaboration has also been proposed by Lisanti's group, who has observed MCT1 in tumors, and MCT4 in stroma, prompting them to characterize this as a “reverse Warburg Effect” (Witkiewicz et al., 2012). These examples highlight alternative roles for MCTs in addition to regulating pH_i_ homeostasis and that inhibition of MCTs may disrupt tumor metabolism homeostasis providing therapeutic benefit.

## pH-sensing proteins that regulate actin assembly play role in cancer cell migration

Although the effects of pH on intracellular processes are ubiquitous, one of the major pH-sensitive systems in the cells is the actin cytoskeleton. The assembly of globular (G-actin) to filamentous (F-actin) and higher structures as well as the reverse process of filament disassembly play different roles in cancer cell processes and behaviors such as vesicle trafficking, contraction, migration, invasion, and metastasis. De novo actin assembly in mammalian cells requires pH_i_ > 7.2 (Webb et al., 2011) and changes (of 0.3–0.4 units) in pH_i_ induce dramatic differences in actin filament assemblies and architectures. These observations suggest that disruption of pH_i_ in either direction can negatively impact the mobility of cancer cells and eventually affect metastasis (Hansen and Kwiatkowski, 2013).

### ADF/Cofilin

Polymerizing actin filaments, which are essential for migratory cells, requires the activity of the actin-severing protein, cofilin (Sidani et al., 2007). The actin depolymerizing factor (ADF)/cofilin family, including ADF, non-muscle cofilin-1 and muscle cofilin-2, are pH sensors that sever and nucleate actin filaments (Bravo-Cordero et al., 2013). In migrating cells, cofilin increases filament disassembly at one end of actin networks and generates free ends for nucleation and assembly at the plasma membrane (Bernstein and Bamburg, 2010). The severing activity of cofilin requires (de)phosphorylation of an N-terminal serine residue and (de-) protonation of a C-terminal histidine residue. Overactivity of cofilin requires both the dephosphorylation of the serine and the deprotonation of histidine to occur. Although cofilin is not directly pH regulated, its binding to Phosphatidylinositol (Lope-Piedrafita et al., 2008) in the plasma membrane is pH-dependent and regulates its activity. Dissociation from membrane PI at more alkaline pH_i_ increases cofilin activity (Frantz et al., 2008).

ADF is extremely pH sensitive and its regulation occurs through a pH-dependent salt-bridge at the F site between His133 and Asp98 of β-strand4. The salt-bridge is stable at slightly acidic conditions but broke at alkaline pH, leading to partial unfolding of the F site and presumably increases actin-binding activity of ADF. Cofilin and ADF have differential pH sensitivity because they bind to actin in different ways (Schonichen et al., 2013b). Cofilin binding to cortactin in invadopodia (i.e., invasive plasmamembrane protrusions of cancer cells) sequesters inactive cofilin at the distal membrane of invadopodia and its binding affinity decreases as pH_i_ increases (Magalhaes et al., 2011).

### Talin

Dynamic remodeling of actin filaments at focal adhesion sites is also pH_i_ sensitive. This regulation is critical at the leading edge of migrating cells where increased pH_i_ is needed (Stock and Schwab, 2009). It has been shown that pH_i_ is higher at the leading edge of migrating cells and it is actually highest at the focal adhesions (Martin et al., 2011; Ludwig et al., 2013). The effect of pH_i_ on focal adhesion is mediated partly by talin binding to F-actin, which binds actin filaments to focal adhesions. The N-terminal domain of talin that binds β-subunits of integrin receptors at the cytosolic side contains a pH-insensitive F-actin-binding site (Critchley, 2009). On the other hand, the C-terminal module that also binds F-actin is pH dependent (Critchley, 2009). So, talin is a pH-sensor whose binding to actin filaments decreases at a pH > 7.2, permitting faster focal adhesion turnover and increased migration (Srivastava et al., 2008). Protonation of amino acids in a pH-sensor region causes conformational changes at distant actin-binding sites and allosterically regulates the actin-binding domain of talin (Srivastava et al., 2008; Critchley, 2009).

### Guanine nucleotide exchange factors

Some guanine nucleotide exchange factors (GEFs) contain PH (plextrin-homology) domains that show binding specificity to membrane phosphoinositides. One example is Dbs, a Dbl family Rho GEF that activates the Rho GTPase Cdc42 at the leading edge of migrating cells that leads to a polarized movement. Dbs contains a PH domain that binds to PIP_2_ with higher affinity at lower pH (Frantz et al., 2007). However, there are some other Dbl family Rho GEFs, such as intersectin, that contain a similar PH domain but with pH-independent binding to PIP_2_. Most of Dbs but not all (such as intersectin) have a histidine (His843) at the PIP_2_-binding site of the pH-sensitive domain (Frantz et al., 2007), similar to pH-dependent binding of cofilin to PIP_2_.

Arf GTPases are localized at acidic endosomes and regulate endosomal trafficking and actin dynamics (Donaldson and Jackson, 2011). As an example, the pH domain of Grp1, a GEF for Arf GTPases, has pH-dependent binding to phosphoinositides. In contrast to Dbs, Grp1 binds PIP_3_ instead of PIP_2_ at endosomal membranes, with a higher binding affinity at lower pH (He et al., 2008, 2011). Grp1's increased binding affinity is because of protonation of His355 located in the loop between β6 and β7 that interacts with the phosphate group 4 of inositol 1,3,4,5-tetrakisphosphate (IP_4_) (Schonichen et al., 2013b).

It has been shown recently that phosphoinositide-binding domains are common in physiological pH sensing proteins such as Fab1p, YOTB, Vac1p, and EEA1 (Lee et al., 2005), ENTH and ANTH. They all show increased phosphoinositide binding affinities at pH values below neutral ~7.2. A common feature of these domains is that they all contain at least one histidine for stereospecific phosphoinositide recognition. Although not experimentally shown yet, proteins containing these domains most probably have membrane localization and pH dependent activity and, if associated with the actin cytoskeleton, could regulate the actin filament dynamics at focal adhesions (Schonichen et al., 2013a,b). Understanding the significance of pH-dependent histidine switches for phosphoinositide can help to understand the role of pH-regulated metastasis.

### Section 2. the extracellular pH

A consequence of pH homeostasis in the face of a high metabolic acid load is the transport of H^+^ into the interstitial space. In tumors that are metabolically active, estimates of the interstitial space (Kep) from dynamic contrast enhanced (DCE) MRI, range from 6 to 12%. Hence there is a 10-fold greater acid load on the extracellular environment compared to intracellular, and this is generally relieved via diffusive transport of mobile buffers from the source, i.e., cells, to the sink, i.e., the vasculature (Schornack and Gillies, 2003). If these diffusion path lengths become long, e.g., greater than 50 microns, then the pH_e_ becomes profoundly acidic. Such conditions are not only encountered in cancer, but in other pathological states, such as ischemia, or following trauma, and mammalian cells have evolved (or retained) sophisticated mechanisms to sense their environmental pH and adjust their behaviors accordingly.

## pH sensors in plasma membrane

Plasma membrane pH sensors in cells are categorized mainly as G-protein coupled receptors (GPCRs) and non-GPCR sensors. Proton-sensing GPCR expression varies by cell type. This subfamily of proton-sensing GPCRs includes GPR4, OGR1 (GPR68), TDAG8 (GPR65), and G2A (GPR132), which have also been identified as receptors for lysolipids; sphingosylphosphorylcholine (SPC), lysophosphatidylcholine (LPC), and psychosine (galactosylsphingosine) (Ludwig et al., 2003). OGR1 is coupled with the PLC/Ca^2+^ signaling pathway through Gq_/11_ proteins. On the other hand, GPR4 and TDAG8 are coupled with the cAMP signaling pathways via Gs. It has recently been observed that activation of TDAG8 with acid pH induces down regulation of c-myc in lymphoma cells (Li et al., 2013). Discovery of the proton-sensing ability of transient receptor potential V1 (TRPV1), a calcium-channel also known as the capsaicin receptor and the vanilloid receptor 1, was a breakthrough in the field of proton-sensing mechanisms, because it showed that non-GPCR proteins can also sense pH (Tomura et al., 2010). Another family of non-GPCR sensors is the acid-sensitive ion channels (ASICs), which include 7 proteins from 4 genes. It has been shown that ASIC1 and ASIC2 are expressed in the central nervous system and are important in pain processing (Wemmie et al., 2013). Understanding pH sensors will provide insight into the molecular basis of pH-dependent phenomena that may help us to understand cancer cell behavior and eventually identify new diagnostics and therapeutics.

### OGR1

Ovarian cancer G protein-coupled receptor 1 (OGR1), previously described as sphingosylphosphorylcholine receptor, is a proton sensing receptor that stimulates inositol phosphate formation. It is inactive at pH_e_ ~7.8 and fully activated at pH_e_ ~6.8 (Ludwig et al., 2003). Using a computational model, Ludwig et al demonstrated that the ability of OGR1 to sense pH resides within histidines located in the extracellular loops. They proposed that acidic pH destabilizes the hydrogen bonds of these histidines and switches the receptor to its active conformation. They also predicted five histidines (H17, H20, H84, H169, and H269) are key players and demonstrated that substituting each histidine with phenylalanine reduces the proton-sensing ability of OGR1 (Ludwig et al., 2003)

It has recently been shown that OGR1 deficiency (*OGR1*^−/−^) in Transgenic Adenocarcinoma of the Mouse Prostate (TRAMP) model reduced tumor formation. The same group also showed similar results in murine melanoma cell xenografts in *OGR1*^−/−^ mice (Yan et al., 2012a,b). OGR1 expression is lower in human metastatic compared with primary prostate cancer tissue. In light of this observation, *OGR1* has been characterized as a new metastasis suppressor gene in prostate cancer cells (Singh et al., 2007). This observation also extends to breast, where Li et al recently showed that OGR1 inhibits cell migration through a Gα12/13 -Rho-Rac1 signaling pathway in MCF7 breast cells (Li et al., 2013). These results suggest that OGR1 may act as a tumor and metastasis suppressor. However, the role of pH and proton-activated signaling in this protein and their relationship to this type of anti-tumor behavior needs to be investigated further.

Interestingly, OGR1 was also shown to be overexpressed in human medulloblastoma, a cancer of neuronal precursor cells. Activation of OGR1 in human medulloblastoma cell line triggers activation of the ERK signaling pathway through proton-mediated Ca^2+^ release in the cytoplasm (Huang et al., 2008). This implies how extracellular acidification can promote cell survival in medulloblastoma. This important finding demonstrates a tumor promoting mechanism, different than the anti-tumor mechanism previously described in prostate cancer and melanoma. Additionally, they demonstrated that only proliferating cells have the ability to translate an acidic pH_e_ into gene transcription, suggesting that the acidic microenvironment provides a survival advantage to proliferating cancer cells over non-transformed cells (Glitsch, 2011). In contrast, Yuan et al. have also recently shown that OGR1-mediated [Ca^2+^] elevation induced apoptosis by activation of calcium-sensitive proteases and their downstream signaling molecules such as Bid, Bax, and caspase-3 in rat endplate chondrocytes (Yuan et al., 2013). Taken together, all these observations imply that transduction of the acid-sensing signal is highly context-dependent, leads to various phenotypes, and depends on the cell-specific genetic history and lineage.

### GPR4

G-protein coupled receptor 4 (GPR4), a close relative of OGR1, also responds to pH changes, but elicits cyclic AMP formation and accumulation. GPR4 plays its pH-sensing role through the G_12/13_-protein/Rho, the G_q_/PLC, and the G_s_-protein/cAMP signaling pathways (Liu et al., 2010). Acid-induced GPR4 activates RhoA, stimulating the formation of actin stress fibers (Castellone et al., 2011). GPR4 is overexpressed in breast, ovarian, colon, liver, and kidney tumors at the mRNA level (Sin et al., 2004).

Osteosarcoma cells and primary human osteoblast precursors exhibit strong pH-dependent inositol phosphate formation and constitutively express GPR4 at their membranes. Tumor growth in GPR4^−/−^ mice is abrogated, because of reduced angiogenesis, suggesting that acidic pH-stimulated GPR4 may regulate endothelial cell growth (Wyder et al., 2011). Some GPR4 inhibitors are under development as antiangiogenesis therapeutics (PCT/EP07/63899). Buffer therapy, described above, might also lead to deprotonation and hence, deactivation of GPR4. Combination therapy using buffer or GPR4 inhibitors also might be considered as a possible therapy against primary and metastatic tumors.

### T-cell death-associated gene 8 (TDAG8)

**Figure 3:**
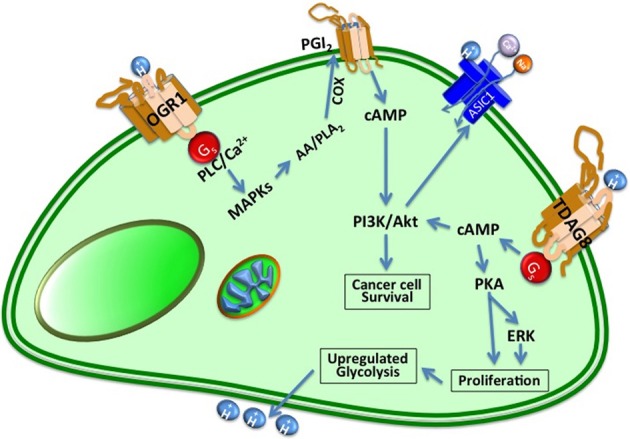
**Cancer cells express proton-sensing GPCRs such as GPR4, TDAG8, and OGR1 to regulate their tumorigenesis and invasion**. TDAG8 and OGR1 sense extracellular protons, leading to activation of the cAMP signaling pathway. OGR1 is usually coupled with the PLC/Ca^2+^ pathway through G_q_/11 proteins and GPR4 and TDAG8 are coupled with the adenylyl cyclase/cAMP pathway through G_s_ proteins in the cells to PKA and ERK signaling pathway that play pivotal roles in cancer progression.

TDAG8 is a pH_e_-sensing GPCR that is overexpressed in different tumor types and cancer cell lines that also might play role in immune response of cancer cells (Ishii et al., 2005). TDAG8, also known as GPR65, was originally identified as an orphan GPCR in apoptotic thymocytes. It was later demonstrated to function as a pH_e_ sensor that responds to extracellular acidification through G_s_, thus, increasing cAMP (Radu et al., 2005). TDAG8 is overexpressed in kidney, colon and ovarian tumors (Sin et al., 2004). TDAG8 can also mediate acid-stimulated cancer bone pain through PKA signaling (Hang et al., 2012). Overexpressed TDAG8 at the cell membrane protects tumor cells against extracellular acidosis and can accelerate tumor development and shRNA knockdown of endogenous TDAG8 attenuates cancer cell survival in acidic media (Ihara et al., 2010). A mechanism for TDAG8 in cancer cells in acidic environment has been proposed wherein cell growth is related to TDAG8-mediated PKA and ERK activation (Figure 3). Overexpression of TDAG8 in NIH3T3 induces different types of oncogenic phenotypes such as rapid proliferation, refractile cell shape, foci formation and tolerance to low serum-conditioned media (Sin et al., 2004). Acid-activated TDAG8 can also stimulate RhoA, actin rearrangement, and stress fiber formation (Ishii et al., 2005).

### Acid-sensitive ion channels (ASICs)

ASICs are voltage-independent ion channels that are mainly expressed in peripheral sensory and central nervous system neurons. They are acid responsive and permeable to cations. They are also modulated by alkalosis and pH_i_ and might play a role of mechanosensation in neurons (Wemmie et al., 2013). These proton-gated cation-selective channels are mostly permeable to Na^+^ ions and belong to the degenerin/epithelial Na^+^ channel (DEG/ENaC) superfamily (Jasti et al., 2007). There are seven subunits of ASICs encoded by four different genes: ASIC1a, ASIC1b1, ASIC1b2, ASIC 2a, ASIC 2b, ASIC 3, and ASIC 4, amongst them 1a, 2a, 2b, and 3 subunits are present in CNS neurons. The low-resolution crystal structure of the chicken ASIC1a at normal and low pH was determined in 2007 and illustrated that functional ASICs are trimeric assemblies (Jasti et al., 2007) (Figure 3). Each ASIC subunit consists of two transmembrane domains (TM1 and TM2) connected with a large extracellular loop that is cysteine rich. ASIC1a and heteromeric ASIC1a/2b channels are permeable to Ca^2+^ and can lead to an accumulation of intracellular Ca^2+^ inside neurons (Sherwood and Askwith, 2008). However, it's been shown that acidosis induced Ca^2+^ accumulation is not only due to the activation of ASIC1a and ASIC1a/2 channels but also could be due to activity of voltage-gated Ca^2+^ channels and/or release of Ca^2+^ from intracellular pools such as sarcoplasmic membranes in cancer cells (Leanza et al., 2013).

ASICs have different roles in peripheral neurons and CNS. In the periphery, it is postulated that ASICs are involved in nociception and mechano-sensation, (Sherwood et al., 2012); and in the CNS, different ASICs play different roles; as ASIC1a is involved in synaptic plasticity, learning/memory and fear conditioning; and ASIC2a is required for the maintenance of retinal integrity and survival of neurons in global ischemia. It has also been described that activation of Ca^2+^-permeable ASIC1a and ASIC1a/2b proteins are involved in acidosis-mediated neuronal degeneration (Pignataro et al., 2011). There is some evidence that ASICs can also play role in cancer progression. ASIC1 and ASIC2 have effects on the growth and migration of glioblastoma cells (Berdiev et al., 2003). Berdiev et al. demonstrated that in grade IV gliomas, glioma cation current is mediated by mixed ASIC1, ASIC2 and inhibition of this conductance decreases glioma growth and cell migration. It has also been shown that knockdown of ASIC1 and ENaC inhibits glioblastoma cell migration (Kapoor et al., 2009). In contrast to ASIC1, increasing surface expression of ASIC2 suppresses the proliferation and migration of GBM cells (Vila-Carriles et al., 2006).

Recently Duan et al also showed that ASIC1 gene deletion suppressed brain derived neurotrophic factor (BDNF) in mice. The BDNF/TrkB pathway enhances ASIC1a ion activity via the phosphoinositide 3-kinase (PI3K)-protein kinase B (PKB/Akt) cascade. PKB/Akt phosphorylates cytoplasmic Ser-25 residue of ASIC1a, resulting in increased trafficking activity and surface expression. By this they proposed a new mechanism for pain hypersensitivity (Duan et al., 2012). ASICs' activity and contribution toward bone-related pain make it a possible target for buffer therapy to neutralize metastatic acid (Nagae et al., 2007; Sluka et al., 2009). In continuation of this observation, a new trial is currently recruiting late-stage cancer patients to further test the ability of buffer therapy to reduce cancer related pain (NCT01846429: Oral Bicarbonate as Adjuvant for Pain Reduction in Patients With Tumor Related Pain, sponsored by H. Lee Moffitt Cancer Center and Research Institute).

### Transient receptor potential channel vanilloid subfamily 1 (TRPV1)

TRPV1 channels are cation channels with a high selectivity for Ca^2+^. Extracellular acidity activates and opens TRPV1 channels in the absence of other stimuli. TRPV1 is not the only proton sensing protein in the family of TRPVs as it has been shown that TRPV4 is also activated by protons in the absence of extracellular Ca^2+^ (Holzer, 2009). TRPV1 is involved in nociception and has been particularly investigated in metastatic bone pain (Kato and Morita, 2013). It has also been reported that the acidic microenvironment of the bone cancer is responsible for the TRPV1-mediated pain. Neurons near metastatic lesions sense the high proton concentration of the tumor environment using these proton-sensing ion channels (Yoneda et al., 2011). TRPV1 is expressed and upregulated in different cancers such as human prostate cancer cells and its activation induces Akt and ERK activation suggesting that TRPV1 activation promotes prostate cancer progression. In contrast, in other reports, activation of TRPV1 leads to induction of apoptosis in cancer cells (Bode and Dong, 2009; Kalogris et al., 2010).

## Conclusions

Historically, the effect of extracellular acidosis had been thought to be transduced via acidification of the pH_i_. However, cells in general, and cancer cells in particular, have redundant and highly active proton exporting mechanisms to maintain the pH_i_ within tight bounds (7.2–7.4) even in the presence of severe extracellular acidosis. An alternative view has emerged over the past decade that the extracellular surface of mammalian cells contains a number of acid-sensors, mediated primarily through expression of surface-accessible histidines. These receptors transduce signals to the cytoplasm and nucleus mediated by cAMP, Ca^2+^, and K^+^, to affect cellular survival, proliferation signaling and cytoskeletal remodeling. Clearly, this is an active and important area of basic research, with implications for normal physiology and pathophysiology.

It is somewhat surprising that there is little evidence for direct mechanistic connectivity between acid sensors and acid transporters. This is particularly true in cancer cells that survive and proliferate in chronically acidic conditions. Acid-sensing proteins affect intracellular signal transduction pathways and gene expression, yet surprisingly none of these appear to directly activate proton extrusion mechanisms. This might suggest that either a low pH_e_ does not contribute greatly to the intracellular acid load or there is a connection that has not yet been elaborated. One proof for the first hypothesis would be that at a nominal membrane potential of −58 mV, the Nernst equation predicts that the equilibrium pH_i_ should be 1.0 pH unit below that of pH_e_. With a reversed pH gradient there is a large proton motive force driving H^+^ into cells. However, even though the permeability of H^+^ to lipid bilayers is orders of magnitude higher than alkali ions, it is still quite low, between 10^−5^–10^−7^ cm s (Nichols and Deamer, 1980; Gutknecht, 1984) implying that the inward directed passive flux of H^+^ across bilayers is much lower than the metabolically produced or outwardly transported flux of H^+^. Consequently, the effect of acidic pH_e_ on the pH_i_ is indirect and thus, it may not be physiologically necessary to couple H^+^ sensing to H^+^ extrusion. Another hypothesis would be that the effect of acidic pH_e_ on the pH_i_ is mediated by highly regulated and coordinated families of carriers. These are most probably electroneutral HCO^−^_3_ transporters since the electoneutral transporters react only to the concentration gradients and not the electromotive force. Uncovering mechanisms of pH_e_ mediation by pH_i_ or vice versa could be an interesting avenue of future investigation in the field of pH sensing and regulation in cancer.

### Conflict of interest statement

The authors declare that the research was conducted in the absence of any commercial or financial relationships that could be construed as a potential conflict of interest.
